# Technological, Nutritional, and Sensory Characteristics of *Gnocchi* Enriched with Hemp Seed Flour

**DOI:** 10.3390/foods11182783

**Published:** 2022-09-09

**Authors:** Maria Merlino, Gianluca Tripodi, Fabrizio Cincotta, Ottavia Prestia, Anthea Miller, Antonio Gattuso, Antonella Verzera, Concetta Condurso

**Affiliations:** 1 Department of Veterinary Sciences, University of Messina, Polo Universitario dell’Annunziata, Viale G. Palatucci, 98168 Messina, Italy; 2 Department of Human Sciences and Promoting of the Quality of Life, San Raffaele Telematic University, Via Val Cannuta 247, 00166 Rome, Italy; 3 Department of AGRARIA, University Mediterranea of Reggio Calabria, via dell’Università 25, 89124 Reggio Calabria, Italy

**Keywords:** hemp seed flour-enriched *gnocchi*, dumpling, source of fiber, texture profile, cooking behavior, volatile profile, sensory analysis, consumer acceptability

## Abstract

Hemp seed flour (HSF) is a by-product of the hemp oil production process and is a valuable source of protein, dietary fiber, minerals, and vitamins. In line with sustainable food production and a circular economy, this research aimed to utilize HSF as fortifying ingredient in the production of *gnocchi*, a typical Italian potato-based fresh pasta, and to investigate the effects of the addition on the quality and consumers’ acceptability of the enriched products. Three formulations have been developed using 5–20% HSF in substitution of soft wheat flour. Nutritional value, cooking quality, color, texture and sensory profile, and the consumers’ acceptability of *gnocchi* samples were evaluated, as well as the functional properties of pure and composite flours and the HSF aroma compounds. HSF addition allowed to enhance the nutritional value of *gnocchi*, gaining the nutritional claim of “source of fiber” in case of formulations with ≥10% of HSF. Moreover, the fortified *gnocchi* had a high technological quality in terms of cooking loss, cooking resistance and textural properties, and average sensory quality; however, the vegetable and hemp odor and the bitter taste make them not well appreciated by consumers highlighting the need for improving the HSF sensory quality for consumers’ satisfaction.

## 1. Introduction

Hemp (*Cannabis sativa* L.) is one of the earliest known cultivated plants in the world, originated in central Asia, and later spread to Europe. It has been widely cultivated and used throughout history for its fiber and afterward for its nutritional and medicinal properties. Several types of research are reported in the literature ranging from traditional use as herbal medicine to the latest potential in COVID-19 [1,2].

Hemp is a multi-purpose crop plant with diverse agricultural and industrial applications including textile, paper, medicine, food, animal feed, paint, biofuel, biodegradable plastic, and construction material; moreover, for its ability to grow under versatile conditions, it is a valuable remedy for polluted soils. Despite this, the presence of the Δ9-tetrahydrocannabinol (Δ9-THC) molecule in the plant has long prohibited its cultivation in several countries but in 2001, the European regulation set the content of Δ9-THC at 0.2% as the maximum level allowed for the cultivation and exploitation of hemp [3,4].

Hemp seeds represent an interesting source of fat (25–35%), mainly polyunsaturated fatty acids (PUFAs) with a high percentage of essential fatty acids, carbohydrates (20–30%), mineral elements, several bioactive molecules, and readily digested proteins (up to 30%) with high amount of essential amino acids [5]. In addition to their high nutritional quality, seeds have also important healthy properties, such as protection against metabolic syndrome, and neurodegenerative and cardiovascular diseases, which are attributed to the high levels of proteins and PUFAs, their composition profile, and to polyphenols and bioactive peptides with powerful antioxidant properties [6].

Hemp seeds, due to the higher lipid content, are mainly used for oil extraction, producing a higher amount of hemp oil cake, from which hemp seed flour (HSF) is obtained. Like seeds, HSF is rich in nutritional and health benefits that make it a valuable product for human consumption Recently HSF was used in the fortification of some backed products [7,8] and durum wheat dry pasta [9] or added to the gluten-free dough for improving bread structure [10]. However, the fast-growing demand for hemp seed oil results in an ever-larger availability of HSF making it necessary to boost the use of this ingredient.

*Gnocchi* is a typical Italian potato-based fresh pasta that can be either homemade or industrially manufactured. Fresh potatoes or potato derivatives, such as flakes, dehydrated products, or flour, constitute the main ingredient to which soft wheat flour, salt, and, sometimes, eggs are added; in the industrial product, emulsifiers, aromas, preservatives, and pasteurized liquid eggs are also included in the recipe [11].

Traditional Italian *gnocchi* is mainly a starchy food and several studies have been carried out for improving its nutritional properties by addition of various fortifying ingredients such as quinoa and amaranth flours [12], green coffee extract [13], navy bean flour and beef meat [14], red rice [15], and buckwheat [15]. To the best of our knowledge, there are no researches on the possibility of using HSF in *gnocchi* dough for enhancing their nutritional quality nor about the effects of hemp flour addition on the color and the technological and sensory quality of fresh pasta.

In line with sustainable food production and a circular economy, the aim of this research was to utilize flour obtained by hemp seed processing side-stream as fortifying ingredient in the production of *gnocchi*, and to investigate the effects on the nutritional, technological and sensory quality, and consumers’ acceptability of the enriched *gnocchi*.

## 2. Materials and Methods

### 2.1. Raw Materials

Type 00 wheat flour (labelled composition: 75.0% carbohydrates, of which 0.5% sugars; 9.0% protein; 1.0% fat, of which 0.2% saturated fat; 2.5% dietary fiber) and dehydrated potatoes (labelled composition: 79.0% carbohydrates, of which 1.0% sugars; 8.0% protein; 0.5% fat; 5.0% dietary fiber; 0.15% sodium) were purchased from the local market. HSF (labelled composition: 26.0% protein; 5.1% carbohydrates, of which 1.8% sugars; 9.6% fat; 46.0% dietary fiber; <0.05% sodium) was provided by Soc. Coop. Molino Crisafulli a.r.l., (Caltagirone, Sicily, Italy) with certification of compliance with Italian legislation on THC content [16]. HSF was made by grinding the hemp (*Cannabis sativa* L. cv. Futura 75) seed cake obtained after the cold pressing extraction of hemp oil.

### 2.2. Functional Properties of HSF, Wheat Flour, and Composite Flours

#### 2.2.1. Bulk Density

For the bulk density determination, 10 g of flour was put into a 100 mL measuring cylinder and tapped to a constant volume [17]. The bulk density was calculated according to the following equation:

$$\mathrm{Bulk}\;\mathrm{density}\left(\frac{g}{{\mathrm{cm}}^{3}}\right)=\frac{\mathrm{flour}\;\mathrm{weight}\;}{\mathrm{flour}\;\mathrm{volume}\;}$$

#### 2.2.2. Water Holding Capacity

Water holding capacity (WHC) was determined as described by Giaimi et al. [18] with slight modifications. 1 g of flour (microwaved at 300 W up to constant weight) was weighed into a falcon test tube, added with 10 mL of distilled water, and vortexed for 30 s. The sample was left to hydrate for 1 h at room temperature. After centrifugation (30 min at 31 g force), the supernatant was removed, and the hydrated sample was weighed.

The water holding capacity was expressed as a percentage and determined according to the following equation:

$$\mathrm{WHC}\;\left(\%\right)=\frac{\left(\mathrm{hydrated}\;\mathrm{flour}\;\mathrm{weight}\;-\mathrm{dry}\;\mathrm{flour}\;\mathrm{weight}\right)}{\mathrm{dry}\;\mathrm{flour}\;\mathrm{weight}}\times 100$$

#### 2.2.3. Swelling Index

The swelling index (SI) was determined as reported by Das et al. [19]. In detail, 2 × 1 g of flour was dispersed in 2 × 10 mL of cold distilled water in two different falcon tube tests. One tube was left to rest for 5 min at room temperature then centrifuged (492× *g* force for 30 min) and the sediment volume was counted as the initial volume. The second tube was immersed in boiling water for 30 min, cooled under running water, centrifuged (492× *g* force for 30 min) and the sediment volume was counted as the final volume. The swelling index was calculated as follows:

$$\mathrm{SI}\;=\frac{\mathrm{final}\;\mathrm{flour}\;\mathrm{volume}}{\mathrm{initial}\;\mathrm{flour}\;\mathrm{volume}}$$

### 2.3. Aroma Compounds of HSF

The aroma compounds of HSF were determined by the HS-SPME-GC-MS technique. For the extraction, 5 g of flour was suspended in 15 mL of saturated sodium chloride solution into a 40 mL vial equipped with a ‘mininert’ valve (Supelco, Bellefonte, PA, USA). Extraction was performed at 35 °C exposing a DVB/CAR/PDMS fiber, 50/30 μm film thickness (Supelco, Bellefonte, PA, USA), to the headspace of the sample for 30 min. The sample was maintained under continuous magnetic stirring and, before extraction, thermally balanced for 30 min. The extracted analytes were directly desorbed into the injector port of the GC/MS held at 260 °C.

For the analysis of the volatile compounds, a Shimadzu GC 2010 Plus gas chromatograph directly interfaced with a TQMS 8040 triple quadrupole mass spectrometer (Shimadzu, Milan, Italy) was used, following the method reported by Condurso et al. [20]. The conditions were: injector temperature, 260 °C; injection mode, splitless; capillary column, VF-WAXms, 60 m × 0.25 mm i.d. × 0.25 µm film thickness (Agilent, S.p.a. Milan, Italy); oven temperature, 45 °C held for 5 min, then increased to 80 °C at a rate of 10 °C/min, to 210 °C at 2 °C/min, and to 250 °C at a rate of 20 °C/min; carrier gas, helium at a constant flow of 1 mL/min; transfer line temperature, 250 °C; acquisition range, 30 to 360 m/z; scan speed, 1250 amu/s. The volatile compounds were identified using mass spectral data, NIST’ 18 (NIST/EPA/NIH Mass Spectra Library, version 2.0, Gaithersburg, MD, USA) and the FFNSC 3.0 database, linear retention indices (LRI), and literature data. Quantitative results were expressed as a percentage of the total peak area.

### 2.4. Dough and Gnocchi Preparation

Three different samples of *gnocchi* fortified with HSF and one control sample (CG) were produced on a laboratory scale according to the traditional Italian recipe. The referenced recipe was made of water, wheat flour, dehydrated potato, and salt; to produce fortified samples, wheat flour was substituted with HSF in different percentages, namely 5% (5HG), 10% (10HG), and 20% (20HG). The formulations used for control and fortified *gnocchi* production are reported in Table 1. The maximum amount of HSF to be used was obtained by means of a preliminary sensorial test with 20 untrained panelists for evaluating consumers’ acceptance of the obtained *gnocchi* (data not shown). The ingredients were thoroughly mixed using a dough mixer (Ariete 1596 Gourmet Professional Metal, De’Longhi Appliances s.r.l, Florence, Italy) for 5 min. All samples were formed by a manual formal *gnocchi* machine (Imperia, Bologna, Italy) and had regular shape and uniform size (1.5 cm × 1.0 cm). For each recipe, three batches were prepared on the same day and six on two different days. In total, 36 samples (nine batches × four recipes) were analyzed, all in triplicate unless otherwise stated in the specific session.

### 2.5. Proximate Chemical Analysis of Gnocchi Samples

Moisture, ash, protein (N × 6.25), and fiber content were determined according to the official methods [21]. The total fat content was determined using a Soxhlet apparatus and hexane as solvent. The carbohydrate content was calculated by difference. *Gnocchi* energy values were determined as the sum of the energy values of lipids, proteins, and carbohydrates, using the following conversion factors: 4 kcal for proteins and carbohydrates; 9 kcal for lipids.

### 2.6. Pasta Cooking Conditions

*Gnocchi* samples were cooked in boiling salted (10 g/L NaCl) mineral water (1:10 *gnocchi*/water ratio). *Gnocchi* samples were cooked until they rose to the water surface (equivalent to the optimal cooking time) and immediately drained for about 30 s just after cooking. The optimal cooking times (OCT) of control and fortified *gnocchi* samples, defined according to preliminary sensory tests (data not shown), were measured using a digital stopwatch (Traceable, VWR International, West Chester, PA, USA) and expressed in seconds.

### 2.7. Weight Increase and Solid Loss

For the determination of weight increase, ten *gnocchi* (~50 g) of each sample were weighed with a precision of 0.01 g, cooked in unsalted water until its optimal cooking time, and drained as described in the “2.6 Pasta Cooking Conditions” session. After draining, *gnocchi* samples were weighed again and the weight gain resulting from cooking was expressed as a percentage (*w*/*w*).

The cooking water has been utilized for the cooking loss determination. In particular, the water was evaporated, and the solid residue dried at 105 °C to constant mass and weighed.

### 2.8. Color

The color analysis was performed using an automatic Minolta CR 300 tristimulus colorimeter (Konica Minolta Business Solutions Italia Spa, Milan, Italy). The CIELAB color system was used as a reference and the following color parameters were measured: L* for brightness [scale: from 0 (black) to 100 (white)], a* [scale: from −50 (green) to +50 (red)], b* [scale: from −50 (blue) to + 50 (yellow)].

The measurement was carried out on both raw and cooked *gnocchi* samples, using each time twelve different *gnocchi* and performing the readings on two different points for each *gnocco*. Following the same procedure, the color of HSF and dehydrated potatoes have been measured, too.

The mean values of L*, a*, b* color parameters were calculated and used to determine the total color difference (Equation (1)) [22].

(1) $${\Delta E}_{Lab}=\sqrt{\Delta L{*}^{2}+\Delta a{*}^{2}+\Delta b{*}^{2}}$$

where ∆*L* = brightness difference, ∆*a* = redness difference, and ∆*b* = yellowness difference.

If 0 < ∆E*_Lab_* < 1 the observer does not notice a difference; 1 < ∆E*_Lab_* < 2 only an experienced observer may notice the difference; 2 < ∆E*_Lab_* < 3.5 an inexperienced observer also notices the difference; 3.5 < ∆E*_Lab_* < 5 a clear difference in color is noticed, and ∆E*_Lab_* > 5 the observer notices two different colors [17].

### 2.9. Texture Analyses

Rheological analyses were performed on doughs, raw and cooked *gnocchi* samples by a TA.XT Plus Texture Analyzer (Stable Micro Systems Ltd., Godalming, UK). Data acquisition and curve integration were carried out by Exponent software 6.1.4.0 (Stable Micro Systems Ltd., Godalming, UK).

The raw and cooked *gnocchi* samples were subjected to the TPA test. The TPA test was performed using a 100 mm compression platen (P/100) probe (Stable Micro Systems Ltd., Godalming, UK) on one *gnocco* sample, both cooked and uncooked, with the following parameters: pre-test speed, 1.00 mm/s; test speed, 5.00 mm/s; post-test speed, 5.00 mm/s; distance: 20.0 mm, trigger force, 5.0 g; data acquisition rate, 400 pps. Twelve replicates were carried out for each sample. The test results expressed different textural characteristics, like hardness, adhesiveness, cohesiveness, gumminess, chewiness, springiness, and resilience.

The doughs were subjected to Stickiness tests. The Stickiness test was performed using a Chen and Hoseney probe (A/DSC) (Stable Micro Systems Ltd., Godalming, UK) on 20 g of dough sample. The probe in contact with the sample measured the forces of insertion and withdrawal from the dough. To evaluate this attribute, the following parameters were used: pre-test speed, 0.50 mm/s; test speed, 0.50 mm/s; post-test speed, 10.00 mm/s; distance, 4.0 mm; trigger force, 5.0 g; data acquisition rate, 400 pps. Twelve replicates were carried out for each dough.

### 2.10. QDA Sensory Analysis

QDA of *gnocchi* samples was carried out according to ISO 13299:2003 [23] using a sensory panel consisting of 8 trained [24] judices (4 males, and 4 females, age range: 23–40 years). The judices were recruited among students and workers of the Department of Veterinary Science of the University of Messina, who reported liking *gnocchi* and habitually consuming them (at least once a month).

All participants signed an informed consent according to the principles of the Declaration of Helsinki before the beginning of the study and they were asked to refrain from smoking, eating, and drinking (excluding water), in the hours before tasting.

The panel was subjected to a 4-week training, during which commercial *gnocchi* and hemp flour of different brands were used to familiarize with the products and develop a common vocabulary to describe unequivocally their perceptions. Globally twenty descriptive terms and their definitions were developed during training. The descriptors were four for the appearance and four for the aroma of raw and cooked *gnocchi*, five for the texture on the touch of raw samples, and, finally, three for the taste, two for the flavor, and seven for the texture in the mouth of cooked *gnocchi*. Reference standards were also developed for each descriptor, corresponding to the highest intensity score on the rating scale used. The descriptors were evaluated using a nine-point intensity scale, where 1 = not perceptible and 9 = strongly perceptible, on a direct computerized registration system (FIZZ Biosystemes. ver. 2.00 M, Couternon, France).

The analyses were carried out in a sensory laboratory according to ISO 8589:2007 [25]. Raw and cooked *gnocchi* were evaluated separately in six different sessions, three for cooked and three for uncooked samples; samples were supplied on white polyethylene dishes labeled with a three-digit random number and served four at a time in randomized order. Raw samples were evaluated for appearance, aroma, and taste, whereas the cooked samples were tested for appearance, aroma, flavor, taste, and texture on the mouth. *Gnocchi* were cooked in salted water (see Pasta Cooking Conditions session) immediately before each tasting session and served as they were. Water was served for cleansing the palate between samples during the cooked *gnocchi* evaluation session. The results were expressed as the average for each sensory attribute.

### 2.11. Consumer’s Acceptability Test

The sensory acceptability of HSF enriched *gnocchi* was evaluated by a panel of 79 untrained judges, thirty-six males and forty-three females, ranging from 22 to 60 years old, recruited from the Department of Veterinary Sciences (University of Messina, Italy). All participants before the beginning of the tasting session signed an informed consent according to the principles of the Declaration of Helsinki.

Each judge evaluated separately raw and cooked *gnocchi* samples in sixty different sessions, thirty for cooked and thirty for uncooked samples. Four samples were evaluated in each session. Panelists were asked to evaluate appearance, odor, and texture of raw *gnocchi*, whereas they were asked to evaluate appearance, odor, taste, and texture on the mouth of cooked samples. *Gnocchi* were cooked in salted water (see Pasta Cooking Conditions session) immediately before each tasting session and served as they were. *Gnocchi* samples were served in randomized order on white plates with randomly coded three-digit codes. Water was served for cleansing the palate between samples during the cooked *gnocchi* evaluation session. A nine-point hedonic scale (1 = dislike extremely, 2 = dislike very much, 3 = dislike moderately, 4 = dislike slightly, 5 = neither like nor dislike, 6 = like slightly, 7 = like moderately, 8 = like very much, and 9 = like extremely) was used by the panelists to consumer’s sensory evaluation [26]. The overall acceptability of *gnocchi* was calculated from the average of all the above sensory parameters. Also, consumers were asked to answer the question “Would you consume this product? with “yes” or “no””.

### 2.12. Statistical Analysis

Excel 2010 software (Microsoft, Milan, Italy) was used to calculate the means and standard deviations on three replicates. The means were analyzed by One-way ANOVA and Duncan’s multiple range test at a confidence level of 95% or more, using the Microsoft XLstat software 2014 (Addinsoft, Paris, France).

## 3. Results and Discussion

The functional properties of wheat flour, HSF, and composite flours are shown in Table 2. The wheat flour (WF) had the highest bulk density, whereas the HSF had the lowest one; as regards the composite flours, 5HSF had the same value as the WF, whereas for 10HSF and 20HSF the bulk density decreased as the HSF percentage increased. The bulk density of flour indicates the weight of a flour sample that can be contained in a fixed volume, and it is affected mainly by particle size and internal porosity. So, the finer granulometry of WF with respect to HSF justifies the variations in bulk density of the flours.

The hydration properties of the analyzed flours were statistically different: HSF showed the highest value of water-holding capacity and WF the lowest one, whereas the composite flours had in-between values with no differences regardless of the HSF percentage. The water-holding property is defined as the ability to absorb water and hold it even after treatment with external forces [27]. Since in a food matrix, water can be retained by hydrogen bonds to other food molecules or by capillary forces, both the chemical composition and the physicochemical characteristics of the flour influence their hydration properties. The high content of HSF dietary fiber justifies the obtained results due to the great number of hydrophilic hydroxyl groups existing in the fiber structure. The WHC can be affected also by the concentration and conformational characteristics of proteins, the degree of interaction of the protein with water, and the relative surface distribution of hydrophilic and hydrophobic amino acids [28].

The flour swelling index showed statistically significant differences among the analyzed samples: the HSF sample had the lowest value whereas the composite flour samples had the highest. The swelling capacity is defined as the volume occupied by a defined amount of hydrated flour and it is affected by the concentration of protein, fiber and, mainly, starch; indeed, the high swelling power in water of starch is further enhanced by the swelling abilities of proteins and fiber [29]. The very low percentage of carbohydrates of HSF with respect to the WF, and the presence of good amounts of starch, along with protein and fiber, in the composite flours account for the differences observed among the analyzed flour samples.

HSF is a good source of proteins, fiber, and unsaturated fat; an increase in the addition of the HSF in the *gnocchi* dough resulted in an increase in the protein, fiber, and fat content of fortified *gnocchi* samples compared to the control sample, as shown in Table 3. In fortified *gnocchi* samples, protein content increased by 4% in 5HG to 16% in 20HG, fat content by 24% in 5HG to 97% in 20HG, and fiber content by 22% in 5HG to 88% in 20HG.

The Regulation (EC) No 1924/2006 [30] of the European Parliament and of the Council of 20 December 2006 on nutrition and health claims made on foods states that the “Source of fiber” nutritional claim, or any claim likely to have the same meaning for the consumer, can be applied to a product containing at least 3 g of fiber per 100 g or at least 1.5 g of fiber per 100 kcal. *Gnocchi* samples enriched with ≥10% of HSF complied with these conditions since 10HG samples contained 1.67 g of fiber per 100 kcal, and 20HG samples contained 2.21 g of fiber per 100 kcal corresponding to 3.73 g of fiber per 100 g. In Europe, a daily fiber intake of 25 g for adults is recommended, with an optimal soluble fiber to insoluble fiber ratio of 1:2 [31]. A portion (220 g) of *gnocchi* with 20% HSF gives about 8 g of fiber that can cover up to about 30% of the daily dietary fiber requirement. While in the hemp flour the total dietary fiber (TDF) is constituted mainly of insoluble fiber (about 80%) [6], potatoes contribute to the TDF of *gnocchi* samples mainly with soluble fiber (about 65% of the TDF in flesh potatoes) [32]. Therefore, *gnocchi* samples fortified with 10% and 20% HSF provide a balanced supply of fiber.

By using the HSF, a significant enhancement of *gnocchi* nutritional value has been achieved: traditional Italian *gnocchi* is mainly a starchy food with modest content of protein and fiber and an irrelevant intake of fat, fortification of *gnocchi* with HSF significantly increases the percentage of all these nutrients. Similarly, other Authors using the by-products of the hemp oil production process (i.e., hemp cake and hemp flour) as fortifying ingredients obtained food products with better nutritional characteristics [7,8,9]. In contrast, previous studies on fortified *gnocchi* revealed that none of the earlier used fortificants allowed to obtain a similar enhancement of *gnocchi* nutritional value; indeed, the use of green coffee extract increased the amount of PUFAs but negatively affected the content of tryptophan, isoleucine and lysine [13], navy bean and meat just increased fat and protein content [14], red rice positively affected fat content but decreased protein level [15], finally buckwheat enhanced fat and fiber content [15].

The cooking behavior of the *gnocchi* samples, i.e., OCT, weight increase, and cooking loss are presented in Table 4. As shown, control and fortified *gnocchi* samples revealed similar OCT values, except for the 20HG sample whose OCT value was the highest (*p* < 0.05). The increase of *gnocchi* weight after cooking was statistically higher for fortified *gnocchi* samples with respect to the control one, with the highest value for 5HG samples and no differences (*p* > 0.05) between 10HG and 20HG samples; this behavior indicates a higher water absorption and retention capability of fortified *gnocchi* in agreement with the WHC and the swelling index values of the composite flours reported in Table 2. Finally, the fortified *gnocchi* samples showed the lowest cooking loss with no differences (*p* > 0.05) among samples with different percentages of HSF. The release of organic materials into the cooking water and the cooking resistance are among the most important parameters for pasta quality assessment. From our data, the cooking loss was always <6%, indicating a good cooking quality of both control and fortified *gnocchi* samples, according to the classification proposed by Hummel [33]; similar results on fortified potato-based pasta have been reported by Cappa et al. [15] and Burgos et al. [12]. As reported in the literature, fortification of pasta with non-traditional ingredients usually weakens the gluten-starch network promoting the leaching of high levels of starch into the cooking water and determining high values of cooking loss [34,35,36,37]. Our results revealed an opposite behavior (cooking loss lower than control) in agreement with previous findings on fortified spaghetti and potato pasta [38,39]. This can be related to the high level of proteins (26%) of the HSF, that can favor network formation with starch, resulting in a compact structure for HSF-enriched *gnocchi* [40]. A more compact structure decreased the rate of water penetration to the core of *gnocchi* accounting for the higher value of OCT of 20HG samples; Teterycz et al. [9] observed similar behavior for pasta enriched with hemp cake and hemp flour and reported the increase in pasta cooking time with the increase in hemp flour and hemp cake addition.

Color is regarded as an important qualitative criterion as it plays a key role in consumer perception and product acceptability. The color measurements (L*, a*, b*) for control and fortified *gnocchi* samples, both raw and cooked, are given in Table 5. According to these results, the incorporation of HSF in *gnocchi* dough led to a significant decrease in lightness (L*), redness (a*), and yellowness (b*) as the percentage of HSF increased; this trend was further enhanced by the cooking process. In detail, the 20HG cooked sample showed the lowest L*, a*, and b* values, whereas the control sample had the highest ones. HSF has a dark greenish-brown color (L* = 21.97 ± 1.09; a* = −3.18 ± 0.15; b*= 7.51 ± 0.23) which is reflected in the color of the fortified *gnocchi* implying the increasing of the greenish color and the masking of the potato yellow color (L* = 86.37 ± 2.86; a*= −0.27 ± 0.05; b*= 15.35 ± 0.51); in addition, the fortified *gnocchi* samples were darker than the control. This agrees with other studies that observed *a* decrease in the L* and b* values due to the addition of hemp flour [7,8].

*Gnocchi* samples fortified with HSF appeared very different in color than the control sample, as indicated by the values of total color difference, ΔE*_Lab_*; ΔE*_Lab_* values ranged between 4.31 (for cooked 5HG) and 10.71 (for raw 20HG) which means that any observer, when looking at the control and fortified samples, notices two fully different colors.

When cooked, the *gnocchi* samples became darker than the corresponding uncooked samples, but they kept their greenish color or even enhanced it, as in the case of the higher percentages of HSF (i.e., 10HSG and 20HSG samples). This suggests that HSF can be successfully employed to confer green color even to the cooked products in agreement with other studies on *gnocchi* fortified with colored flours that reported color keeping of the fortified samples after cooking [15].

Textural parameters, such as hardness, adhesiveness, springiness, cohesiveness, gumminess, chewiness, and resilience of both raw and cooked samples are presented in Table 6. 

As regards raw samples, *gnocchi* enriched with HSF showed statistically significant higher values of hardness, gumminess, and chewiness than the control sample, regardless of the level of HSF addition; on the contrary, the highest (*p* < 0.05) springiness value was observed in the control sample and the lowest ones in 10HG and 20HG samples; finally, no statistically significant differences were noticed among raw samples for adhesiveness, cohesiveness, and resilience.

Concerning cooked *gnocchi*, 10HG and 20HG samples were significantly (*p* < 0.05) harder, gummier, and chewier than the others; instead, adhesiveness, springiness, cohesiveness, and resilience values did not show any statistically significant differences among *gnocchi* samples. The cooking process determined the increase (*p* < 0.05) of hardness, cohesiveness, gumminess, and chewiness and the decrease (*p* < 0.05) of adhesiveness and resilience, whereas no statical differences were observed between raw and cooked samples for springiness.

The hardness of food is strictly correlated to the internal structures of the food product; it has been reported that the firmness of cooked pasta increases as the protein level increases [41,42], and indeed the addition of HSF to *gnocchi* dough enhanced the protein level of the *gnocchi* samples and thus their firmness. Similarly, as reported by Teterycz et al. [9], the hardness of the hemp flour fortified pasta increases in the hemp flour addition range from 5 to 25%. The hardness values of the cooked *gnocchi* fortified with ≥10% HSF were very similar to those reported by Krishnan et al. [38] for sweet potato pasta enriched with 20% dietary fiber. Gumminess and chewiness of raw and cooked *gnocchi* samples showed the same trend of hardness as already reported for fusilli pasta enriched with hemp seed by-products (i.e., flour and cake) [9]; however, the values observed in *gnocchi* samples for these texture parameters were lower than those reported for fusilli pasta probably due to the higher moisture content of fresh pasta (*gnocchi*) with respect to dried pasta (fusilli).

Adhesiveness is a measure of the stickiness of the product while eating and it is related to the amount of starch granules that exudates from the pasta matrix into the cooking water and coat the surface of the product [43]. Adhesiveness is, along with hardness, the most important quality parameter for Italian consumers who require that pasta must be firm to the bite and not become sticky during chewing. Our results revealed, for both control and fortified *gnocchi* samples, adhesiveness values lower than those reported in the literature for fresh potato pasta and fiber-enriched potato pasta [11,38]. Cohesiveness and springiness parameters indicated how the sample holds together upon cooking and, in contrast to that reported for *gnocchi* enriched with rice or buckwheat flours [15] and for pasta fortified with HSF or hemp seed cake [9], these parameters were not detrimentally affected by the addition of HSF to *gnocchi* dough. This indicates that, despite its high fiber content, the HSF was not able to disturb the structure of pasta samples suggesting the use of well-balanced formulations in fortified *gnocchi* manufacture.

The results of the dough stickiness test, shown in Table 7, highlighted that the addition of HSF to *gnocchi* dough reduced the stickiness and the force of adhesion, regardless the of levels of addition; no statistically significant differences were observed for the dough strength/cohesiveness ratio.

Stickiness is described as the force of adhesion that occurs during the contact of two surfaces with each other. Stickiness is a combination of adhesion, the interaction between a material and a surface, and cohesion, the interactions within the material. Dough stickiness is of great importance in food industries; indeed, sticky dough adheres to handling equipment causing difficulties during processing, with interruptions in production, extensive waste, and contamination of machinery. Dough stickiness is influenced by many factors: water absorption capacity and the water-soluble protein levels of flours are positively correlated to dough stickiness, whereas the opposite was observed for granule size and starch levels of the flours [44]. The use of HSF as a fortifying ingredient in potato-based fresh pasta resulted in the enhancement of the surface-related qualities of doughs, probably related to the largest grind size of HSF with respect to wheat flour.

Table 8 reports the percentage composition of the volatile fraction of HSF. 84 volatiles were overall identified; among these, esters, alcohols, acids, ketones, aldehydes, sulfur compounds, hydrocarbons, nitriles, and furanic compounds were determined.

The volatile profile of HSF was constituted mainly by alcohols, aldehydes, and furanic compounds that altogether accounted for 72% of the whole volatile fraction. The main constituents were 2-pentyl furan, hexanal, 3-methyl-1-butanol, 1-hexanol, 3-methylbutanal, 2-ethyfuran, and acetic and hexanoic acid. To the best of our knowledge non information is reported in the literature on the volatile profile of HSF. However, most of the volatile compounds here identified in HSF are secondary metabolites of the plant (Cannabis sativa L) that arise from the different biosynthetic pathways occurring in the vegetable tissues. Through the lipoxygenase pathway, aldehydes, alcohols, acids, and esters containing 6 to 10 carbon atoms are synthesized from unsaturated fatty acids; the so-called green leaf volatiles, i.e., C6 aldehydes, alcohols, and their ester, arise from linoleic and linolenic acids that are the main fatty acids in hemp seeds. Branched-chain aldehydes, alcohols, acids, and esters originate from branched-chain amino acid degradation: compounds containing the 3-methylbutyl moieties and 3-methylbutanoate esters from leucine; 2-methyl propyl compounds and 2-methylpropanoate esters from valine; 2-methylbutyl compounds and 2-methylbutanoate esters from isoleucine [45]. 2-Akylfurans, instead, originate from polyunsaturated fatty acid autoxidation and photosensitized oxidation, with 2-pentylfuran being the decomposition product of linoleate hydroperoxides [46]; their presence in HSF denotes unsuitable packaging and storage conditions.

**Figure 1:**
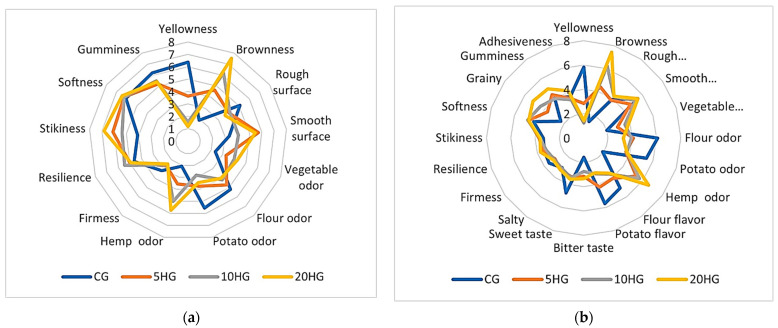
Spider plot representation of the QDA sensory analysis of control and hemp seed flour-enriched *gnocchi* samples: (**a**) raw samples; (**b**) cooked samples.

For the sensory quality assessment, trained panelists were asked to evaluate different attributes regarding color, appearance, odor, and texture to the touch for the raw samples and appearance, odor, flavor, taste, and texture for the cooked samples (Figure 1). According to the panelist evaluations, fortified *gnocchi*, both raw and cooked, significantly differed from the control for the higher vegetable and hemp odor, the lower potato odor, the higher brownness, and the lower yellowness. When considering the cooked samples, fortified *gnocchi* tasted less sweet and more bitter than the control and had a lower potato and flour flavor. As regards the texture attributes, panelists judged the fortified *gnocchi* just less firm and grainier than the control and stickier to the touch.

**Figure 2:**
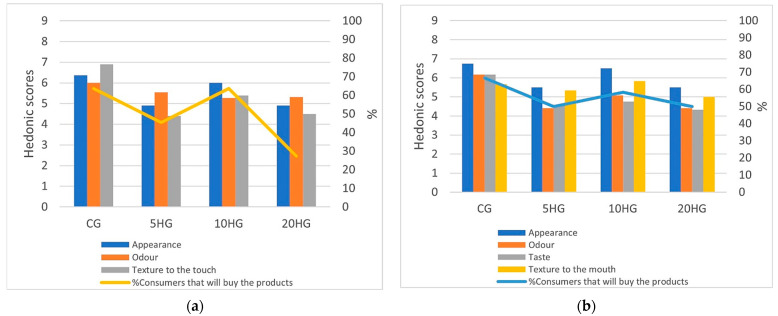
Combo chart representation of the hedonic consumer test for control and hemp seed flour-enriched *gnocchi* samples: (**a**) raw samples; (**b**) cooked samples.

The consumer panel assessed the *gnocchi* acceptability by evaluating odor, appearance, and texture to the touch for raw samples and odor, appearance, taste, and texture for the cooked samples. The lowest scores were recorded for taste, odor, and texture to the touch for the raw and cooked 5HG and 20HG samples (Figure 2). However, these values were never inferior to 4 indicating just a slight disliking. The 10HG samples were the most appreciated among fortified samples with odor, appearance, and texture scores between 5.08 and 6.50, whereas the taste score remained inferior to 5 also for this sample. The results clearly indicated that the taste of hemp-enriched *gnocchi* did not meet consumers’ satisfaction due to the bitter taste of the vegetable flour that detrimentally affected *gnocchi* acceptability regardless of the formulation. When considering the percentage of consumers willing to buy the fortified *gnocchi*, values ≥50% were noted for all cooked samples, whereas the 20HG raw samples showed the lowest value.

Literature data indicate that fortified *gnocchi* do not usually gain great consumer acceptability. Indeed, the acceptability and purchase/consumption intentions of *gnocchi* fortified with amaranth flour [12] or quinoa flour [12] were lower than conventional *gnocchi*, and even lower than those gained by our fortified samples. Cappa et al. [15] also reported that *gnocchi* fortified with red rice flour or buckwheat flour were judged of poor sensory quality and rejected by consumers. Only in the case of *gnocchi* enriched with navy beans and beef meat [14], the authors while not showing the data referred that the added ingredients did not affect the consumer overall liking of the products.

## 4. Conclusions

In this study, HSF, a by-product of the hemp oil production process obtained by grinding hemp cake, has been characterized for its functional properties and volatile aroma profile and used as a fortifying ingredient to produce *gnocchi*, a typical Italian potato-based fresh pasta.

The results of this study revealed good hydration properties of the HSF and its ability to enhance the swelling properties of soft WF. The HSF volatile profile was dominated by the so-called green leaf volatiles and 2-alkylfurans, both deriving from the oxidative breakdown of the major hemp seed fatty acids.

As regards *gnocchi*, this study clearly demonstrated that the HSF can be successfully used to improve the nutritional value of fresh pasta enhancing fat, protein, ash and fiber content and reducing carbohydrate amount. Using the formulations enriched with ≥10% of HSF, fresh pasta responding to the claim “source of fibers” was realized, confirming the potential of HSF as a very nutritious ingredient. None of the earlier used fortifying ingredients allowed to obtain such full enhancement of *gnocchi* nutritional value. Moreover, HSF showed to have a good texturizing capacity resulting in a lower stickiness of the *gnocchi* dough, and in fortified *gnocchi* samples with low cooking loss, good cooking resistance and firmness, and low stickiness. The color analysis suggested that the addition of HSF to the dough can confer a greenish color to fortified *gnocchi*, which is kept even after cooking. Unfortunately, from a sensory point of view, the fortified *gnocchi* revealed a bitter taste and a vegetable and hemp odor that made them not well appreciated by consumers. Further studies are necessary for the assessment of new strategies aiming to improve the sensory quality of HSF and, thus, overcome the sensory defects of hemp seed flour-enriched products, so meeting consumers’ satisfaction.

## Figures and Tables

**Table 1:** Control and hemp seed flour-enriched *gnocchi* formulations.

Ingredients (g/100 g)	CG ^1^	5HG ^2^	10HG ^3^	20HG ^4^
Water	49.85 ± 0.01	49.85 ± 0.01	49.85 ± 0.01	49.85 ± 0.01
Dehydrated potato	29.91 ± 0.01	29.91 ± 0.01	29.91 ± 0.01	29.91 ± 0.01
Wheat flour	19.94 ± 0.01	18.94 ± 0.01	17.95 ± 0.01	15.95 ± 0.01
HSF	-	1.00 ± 0.01	1.99 ± 0.01	3.99 ± 0.01
Salt	0.29 ± 0.01	0.30 ± 0.01	0.30 ± 0.01	0.30 ± 0.01

^1^ Control *gnocchi* sample; ^2^ *gnocchi* samples fortified with 5% HSF; ^3^ *gnocchi* samples fortified with 10% HSF; ^4^ *gnocchi* samples fortified with 20% HSF.

**Table 2:** Functional properties of wheat flour, hemp seed flour, and composite flours.

	WF ^1^	HSF ^2^	5HSF ^3^	10HSF ^4^	20HSF ^5^
Bulk density (g/mL)	0.71 ± 0.01 a ^6^	0.60 ± 0.01 c	0.71 ± 0.01 a	0.69 ± 0.01 b	0.67 ± 0.01 b
Water holding capacity (%)	93.12 ± 0.009 c	181.6 ± 0.007 a	101.098 ± 0.007 b	100.8 ± 0.009 b	103.01 ± 0.005 b
Swelling index	3.3 ± 0.11 b	1.4 ± 0.05 c	4.0 ± 0.10 a	3.7 ± 0.08 a	3.9 ± 0.08 a

^1^ 100% wheat flour 00 type; ^2^ 100% hemp seed flour; ^3^ 5% hemp seed flour + 95% wheat flour; ^4^ 10% hemp seed flour + 90% wheat flour; ^5^ 20% hemp seed flour + 80% wheat flour. ^6^ Data are expressed as the mean ± standard deviation. Different letters in the same row indicate significant differences at *p* < 0.05 among samples.

**Table 3:** Nutrient content (%) of control and hemp seed flour-enriched *gnocchi* samples.

	CG ^1^	5HG ^2^	10HG ^3^	20HG ^4^
Moisture	54.54 ± 0.63	54.55 ± 0.60	54.56 ± 0.61	54.57 ± 0.58
Protein	4.19 ± 0.03 d ^5^	4.36 ± 0.01 c	4.53 ± 0.04 b	4.87 ± 0.08 a
Carbohydrates	38.58 ± 0.39 a	37.89 ± 0.41 b	37.19 ± 0.40 b	35.80 ± 0.36 c
Fiber	1.99 ± 0.01 d	2.43 ± 0.02 c	2.86 ± 0.02 b	3.73 ± 0.02 a
Fat	0.35 ± 0.00 d	0.43 ± 0.00 c	0.52 ± 0.00 b	0.69 ± 0.00 a
Ash	0.33 ± 0.00 b	0.34 ± 0.00 a	0.34 ± 0.00 a	0.34 ± 0.00 a
Kcal	174 ± 0.78 a	173 ± 0.82 b	172 ± 0.80 b	169 ± 0.72 c

^1^ Control *gnocchi* sample; ^2^ *gnocchi* samples fortified with 5% HSF; ^3^ *gnocchi* samples fortified with 10% HSF; ^4^ *gnocchi* samples fortified with 20% HSF. ^5^ Data are expressed as the mean ± standard deviation. Different letters in the same row indicate significant differences at *p* < 0.05 among samples.

**Table 4:** Cooking quality parameters of control and hemp seed flour-enriched *gnocchi* samples.

	CG ^1^	5HG ^2^	10HG ^3^	20HG ^4^
OCT ^5^/s	98 ± 4b ^6^	107 ± 8 b	107 ± 7 b	124 ± 5 a
Weight increase (% *w*/*w*)	18.40 ± 1.71 c	25.97 ± 3.05 a	22.38 ± 2.98 b	21.61 ± 2.84 b
Cooking loss (% *w*/*w*)	5.58 ± 0.74 a	3.10 ± 0.21 b	2.92 ± 0.23 b	2.82 ± 0.21 b

^1^ Control *gnocchi* sample; ^2^ *gnocchi* samples fortified with 5% HSF; ^3^ *gnocchi* samples fortified with 10% HSF; ^4^ *gnocchi* samples fortified with 20% HSF; ^5^ Optimal Cooking Time. ^6^ Data are expressed as the mean ± standard deviation. Different letters in the same row indicate significant differences at *p* < 0.05 among samples.

**Table 5:** Color profile of control and hemp seed flour-enriched *gnocchi* samples.

	CG ^1^			5HG ^2^			10HG ^3^			20HG ^4^		
	Raw	Cooked	Raw vs. Cooked	Raw	Cooked	Raw vs. Cooked	Raw	Cooked	Raw vs. Cooked	Raw	Cooked	Raw vs. Cooked
L* (D65)	74.62 ± 1.72 a	67.58 ± 2.75 A	** ^5^	68.91 ± 3.38 b	64.81 ± 3.64 B	**	69.19 ± 5.45 b	60.78 ± 2.77 C	**	64.9 ± 4.09 c	57.77 ± 2.9 D	**
a* (D65)	−0.46 ± 0.14 a	−0.34 ± 0.41 A	ns ^6^	−0.95 ± 0.19 b	−0.76 ± 0.33 B	**	−1.24 ± 0.42 c	−1.97 ± 0.38 C	*	−1.33 ± 0.24 c	−2.71 ± 0.28 D	**
b* (D65)	15.74 ± 0.81 a	10.17 ± 1.62 B	**	15.32 ± 1.82 a	13.44 ± 1.95 A	**	14.21 ± 1.27 b	12.85 ± 0.77 A	**	11.33 ± 1.06 c	11.14 ± 1.33 B	ns
ΔE_Lab_	-	-		5.75 ± 0.62 b	4.31 ± 0.82 C	*	5.70 ± 0.36 b	7.49 ± 0.52 B	**	10.71 ± 0.98 a	10.14 ± 0.81 A	ns

^1^ Control *gnocchi* sample; ^2^ *gnocchi* samples fortified with 5% HSF; ^3^ *gnocchi* samples fortified with 10% HSF; ^4^ *gnocchi* samples fortified with 20% HSF. Data are expressed as the mean ± standard deviation. Different case letters in the same row indicate significant differences at *p* < 0.05 among raw samples. Different capital letters in the same row represent significant differences at *p* < 0.05 by Duncan’s multiple range test among cooked samples. ^5^ Statistically significant differences between raw and cooked samples at *p* < 0.01 (**) or *p* < 0.05 (*); ^6^ not statistically significant (*p* > 0.05).

**Table 6:** Texture parameters of control and hemp seed flour-enriched *gnocchi* samples.

	CG ^1^			5HG ^2^			10HG ^3^			20HG ^4^		
	Raw	Cooked	Raw vs. Cooked	Raw	Cooked	Raw vs. Cooked	Raw	Cooked	Raw vs. Cooked	Raw	Cooked	Raw vs. Cooked
Hardness/N	nd ^5^ b	0.98 ± 0.16 B	** ^6^	0.60 ± 0.06 a	0.88 ± 0.18 B	**	0.65 ± 0.05 a	1.41 ± 0.35 A	**	0.63 ± 0.09 a	1.36 ± 0.26 A	**
Adhesiveness/Ns	nd	−0.02 ± 0.02	ns ^7^	−0.01 ± 00	−0.09 ± 0.07	**	n.d.	−0.05 ± 0.02	**	−0.01 ± 0.01	−0.02 ± 0.02	*
Springiness ^8^	0.98 ± 0.04 a	1.11 ± 0.29	ns	0.95 ± 0.02 ab	1 ± 0.05	*	0.93 ± 0.02 b	0.89 ± 0.31	ns	0.92 ± 0.03 b	0.98 ± 0.02	**
Cohesiveness ^8^	0.82 ± 0.06	0.89 ± 0.02	**	0.81 ± 0.01	0.88 ± 0.03	**	0.79 ± 0.02	0.80 ± 0.3	ns	0.79 ± 0.03	0.86 ± 0.02	**
Gumminess/N	nd b	0.89 ± 0.15 B	**	0.52 ± 0.11 a	0.77 ± 0.17 B	*	0.53 ± 0.07 a	1.27 ± 0.33 A	**	0.50 ± 0.08 a	0.92 ± 0.23 A	**
Chewiness/N	nd b	0.74 ± 0.28 B	**	0.49 ± 0.11 a	0.76 ± 0.18 B	*	0.50 ± 0.08 a	1.24 ± 0.32 A	**	0.46 ± 0.09 a	0.90 ± 0.23 A	**
Resilience ^8^	0.39 ± 0.03	0.01 ± 00	**	0.40 ± 0.02	0.01 ± 00	**	0.37 ± 0.04	0.01 ± 00	**	0.40 ± 0.02	0.01 ± 00	**

^1^ Control *gnocchi* sample; ^2^ *gnocchi* samples fortified with 5% HSF; ^3^ *gnocchi* samples fortified with 10% HSF; ^4^ *gnocchi* samples fortified with 20% HSF. ^5^ not detected. Data are expressed as the mean ± standard deviation. Different case letters in the same row indicate significant differences at *p* < 0.05 among raw samples. Different capital letters in the same row represent significant differences at *p* < 0.05 by Duncan’s multiple range test among cooked samples. ^6^ Statistically significant differences between raw and cooked samples at *p* < 0.01 (**) or *p* < 0.05 (*); ^7^ not statistically significant (*p* > 0.05). ^8^ dimensionless parameters.

**Table 7:** Surface-related qualities of control and hemp seed flour-containing doughs.

	CD ^1^	5HD ^2^	10HD ^3^	20HD ^4^
Stickiness (N)	0.32 ± 0.02 a ^5^	0.26 ± 0.01 b	0.26 ± 0.02 b	0.26 ± 0.02 b
Work of Adhesion (Ns)	0.03 ± 0.01 a	0.02 ± 0.00 b	0.02 ± 000 b	0.01 ± 0.00 b
Dough Strength/Cohesiveness (mm)	1.48 ± 0.26	1.26 ± 0.14	1.12 ± 0.16	1.20 ± 0.76

^1^ Control *gnocchi* dough; ^2^ *gnocchi* dough containing 5% HSF; ^3^ *gnocchi* dough containing 10% HSF; ^4^ *gnocchi* dough containing 20% HSF. ^5^ Data are expressed as the mean ± standard deviation. Different letters in the same row indicate significant differences at *p* < 0.05 among dough samples.

**Table 8:** Volatile fraction composition of hemp seed flour.

Volatile Compounds	LRI ^1^	Percentage ^2^	Volatile Compounds	LRI ^1^	Percentage ^2^
**Acids**			**Esters**		
Acetic acid	1450	3.30 ± 0.29	Ethyl acetate	883	0.33 ± 0.02
Propanoic acid	1536	0.11 ± 0.00	Propyl acetate	971	0.15 ± 0.01
3-Methylbutanoic acid	1666	0.35 ± 0.02	3-Methylbutyl acetate	1117	0.30 ± 0.02
Pentanoic acid	1733	0.21 ± 0.02	Hexyl acetate	1266	0.07 ± 0.00
(*E*)-2-Butenoic acid	1772	0.19 ± 0.01	γ-Butyrolactone	1629	0.41 ± 0.03
Hexanoic acid	1839	2.52 ± 0.19	γ-Hexalactone	1699	0.40 ± 0.03
Heptanoic acid	1945	0.12 ± 0.00	γ-Nonalactone	2020	0.12 ± 0.01
Octanoic acid	2051	0.19 ± 0.01	All		1.78 ± 0.05
Nonanoic acid	2156	0.28 ± 0.01	**Ketones**		
2-Octenoic acid	2175	0.12 ± 0.00	Acetone	811	0.44 ± 0.03
Decanoic acid	2262	0.20 ± 0.01	2-Butanone	900	0.38 ± 0.02
All		7.59 ± 0.35	Heptan-2-one	1178	2.88 ± 0.19
**Aldehydes**			3-Octanone	1249	0.36 ± 0.02
2-Methylbutanal	911	1.50 ± 0.10	2-Octanone	1281	1.48 ± 0.09
3-Methylbutanal	915	5.94 ± 0.32	2-Methyl-1-hepten-6-one	1328	0.11 ± 0.00
Pentanal	976	2.24 ± 0.12	6-Methyl-5-hepten-2-one	1333	0.29 ± 0.01
Hexanal	1078	10.67 ± 0.96	3-Octen-2-one	1405	0.35 ± 0.02
2-Methyl-2-butenal	1094	0.17 ± 0.01	3,5-Octadien-2-one	1516	0.38 ± 0.02
Heptanal	1182	0.21 ± 0.02	All		6.68 ± 0.22
(*E*)-2-Hexenal	1217	0.59 ± 0.04	**Terpenoids**		
Octanal	1286	0.08 ± 0.00	α-Pinene	1014	1.50 ± 0.08
(*Z*)-2-Heptenal	1323	1.47 ± 0.09	Camphene	1058	0.12 ± 0.01
(*E*)-2-Octenal	1427	0.30 ± 0.02	β-Pinene	1103	0.42 ± 0.03
(*E,E*)-2,4-Heptadienal	1492	0.47 ± 0.02	3-Carene	1144	0.68 ± 0.04
Benzaldehyde	1523	1.30 ± 0.09	β-Myrcene	1157	0.41 ± 0.03
(*E*)-2-Nonenal	1532	0.50 ± 0.02	Eucalyptol	1206	0.26 ± 0.02
All		25.45 ± 1.03	p-Cymene	1268	0.37 ± 0.02
**Alcohols**			p-Cymenene	1435	0.46 ± 0.03
2-Methyl-1-propanol	1082	1.69 ± 0.10	Linalool	1539	0.08 ± 0.00
2-Pentanol	1110	0.10 ± 0.00	(*Z*)-Pinocamphone	1543	0.15 ± 0.01
1-Butanol	1133	0.23 ± 0.01	Fenchyl alcohol	1576	0.12 ± 0.01
1-Penten-3-ol	1149	0.87 ± 0.06	(*E*)-α-Bergamotene	1580	0.27 ± 0.02
3-Methyl-1-butanol	1198	10.11 ± 0.99	Caryophyllene	1592	2.00 ± 0.11
1-Pentanol	1239	1.81 ± 0.11	Terpinen-4-ol	1596	0.10 ± 0.00
(*Z*)-2-Penten-1-ol	1305	0.08 ± 0.00	(*E*)-Pinocarveol	1648	0.19 ± 0.02
2-Heptanol	1309	0.52 ± 0.03	(*E*)-β-Farnesene	1658	0.13 ± 0.00
(*E*)-2-Penten-1-ol	1312	0.07 ± 0.00	α-Humulene	1663	0.56 ± 0.03
1-Hexanol	1343	9.98 ± 0.75	Neral	1680	0.92 ± 0.06
(*Z*)-2-Hexen-1-ol	1399	0.05 ± 0.00	α-Terpineol	1689	0.09 ± 0.00
2-Octanol	1410	0.12 ± 0.00	endo-Borneol	1693	0.06 ± 0.00
1-Octen-3-ol	1440	0.55 ± 0.03	β-Selinene	1711	0.17 ± 0.01
1-Heptanol	1446	0.29 ± 0.01	α-Selinene	1717	0.07 ± 0.00
(*Z*)-2-Octen-1-ol	1608	0.13 ± 0.01	Caryophyllene oxide	1971	0.17 ± 0.01
1-Nonanol	1651	0.06 ± 0.00	All		9.30 ± 0.17
Benzyl alcohol	1869	0.05 ± 0.00	**Nitriles**		
Phenyl ethyl alcohol	1903	0.12 ± 0.00	Hexanenitrile	1298	0.60 ± 0.03
1-Dodecanol	1956	0.08 ± 0.00	All		0.60 ± 0.03
1-Tetradecanol	2160	0.13 ± 0.01	**Furanic compounds**		
All		27.05 ± 1.39	2-Methylfuran	864	0.59 ± 0.04
**Hydrocarbons**			2-Ethylfuran	950	4.13 ± 0.29
3-Methylnonane	957	0.22 ± 0.01	2-Propylfuran	1027	0.35 ± 0.02
Decane	991	0.93 ± 0.06	2- Butylfuran	1128	1.12 ± 0.09
Dodecane	1190	0.55 ± 0.03	2-Pentylfuran	1226	13.34 ± 1.01
Tridecane	1290	0.32 ± 0.02	All		19.53 ± 1.06
All		2.02 ± 0.07			

^1^ Linear retention indexes calculated according to the Van Den Dool and Kratz equation; ^2^ percentage of peak area in TIC chromatogram.

## Data Availability

Data is contained within the article.
